# Royal Medical Benevolent College, Epsom

**Published:** 1890-04-26

**Authors:** C. Holman

**Affiliations:** Treasurer of Epsom College. Reigate


### Editor's Letter-Box.

### ROYAL MEDICAL BENEVOLENT COLLEGE, EPSOM.

Sir,—I have the pleasure to send you a list of the contributions collected at the festival on April 17th, in aid of the fund for the Pensioners and Foundation Scholars of Epsom College. The Council are most grateful for the liberal response made to the eloquent appeal of Sir James Paget, but in consequence of the representations of some warm friends of the institution, it has been determined to keep the list open until the annual general meeting of the Governors, on May 22nd. Any contributions may be sent to the office, 37, Soho Square, W.—Your faithful servant,

C. Holman,
Treasurer of Epsom College.

Reigate, April 21st, 1890.

